# Assessing trends in population size of three unmarked species: A comparison of a multi‐species N‐mixture model and random encounter models

**DOI:** 10.1002/ece3.10595

**Published:** 2023-10-13

**Authors:** Martijn Bollen, Pablo Palencia, Joaquín Vicente, Pelayo Acevedo, Lucía Del Río, Thomas Neyens, Natalie Beenaerts, Jim Casaer

**Affiliations:** ^1^ Centre for Environmental Sciences UHasselt – Hasselt University Diepenbeek Belgium; ^2^ Data Science Institute UHasselt – Hasselt University Diepenbeek Belgium; ^3^ Research Institute for Nature and Forest Brussels Belgium; ^4^ Instituto de Investigación en Recursos Cinegéticos (IREC) CSIC‐ UCLM‐ JCCM Ciudad Real Spain; ^5^ Dipartamiento di Scienze Veterinarie Università Degli Studi di Torino Grugliasco Torino Italy; ^6^ Leuven Biostatistics and statistical Bioinformatics Centre KU Leuven Leuven Belgium

**Keywords:** abundance, camera trapping, N‐mixture model, population trends, random encounter model, unmarked

## Abstract

Estimation of changes in abundances and densities is essential for the research, management, and conservation of animal populations. Recently, technological advances have facilitated the surveillance of animal populations through the adoption of passive sensors, such as camera traps (CT). Several methods, including the random encounter model (REM), have been developed for estimating densities of unmarked populations but require additional information. Hierarchical abundance models, such as the N‐mixture model (NMM), can estimate abundances without performing additional fieldwork but do not explicitly estimate the area effectively sampled. This obscures the interpretation of its densities and requires its users to focus on relative measures of abundance instead. Hence, the main objective of our study is to evaluate if REM and NMM yield consistent results qualitatively. Therefore, we compare relative trends: (i) between species, (ii) between years and (iii) across years obtained from annual density/abundance estimates of three species (fox, wild boar and red deer) in central Spain monitored by a camera trapping network for five consecutive winter periods. We reveal that NMM and REM provided density estimates in the same order of magnitude for wild boar, but not for foxes and red deer. Assuming a Poisson detection process in the NMM was important to control for inflation of abundance estimates for frequently detected species. Both methods consistently ranked density/abundance across species (between species trend), but did not always agree on relative ranks of yearly estimates within a single population (between years trend), nor on its linear population trends across years (across years trend). Our results suggest that relative trends are generally consistent when the range of variability is large, but can become inconsistent when the range of variability is smaller.

## Graphical abstract

Comparing relative trends in abundance/density between random encounter models and N‐mixture models for red fox, wild boar and red deer populations. The methods agree on species ranking of abundance/density but do not always agree on relative ranks of yearly estimates within a single population, nor on its linear population trends.

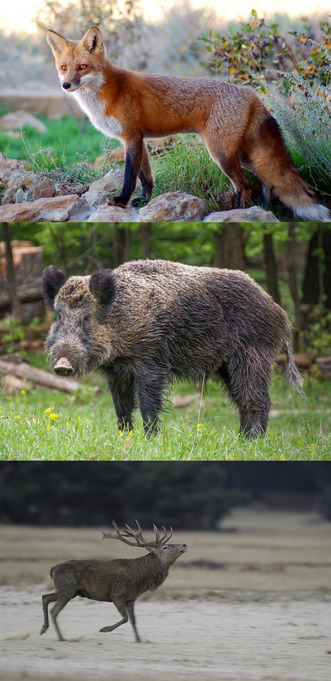

## 1 INTRODUCTION

Obtaining reliable demographic parameters, including (age‐specific) survival, immigration, fecundity, and population size, is essential in wildlife management (Carpio et al., 2021; Williams et al., 2002). Since the estimation of population size over time enables population monitoring and is cheap in terms of data requirements (i.e., counts from point surveys replicated in space and time are sufficient), it is the (main) parameter of interest in many ecological studies. Moreover, collecting population counts has become increasingly cost‐efficient over the last decades due to the adoption of automated sensor networks, such as camera traps (CT). Analytical frameworks for counts can yield precise estimates of population size (Keever et al., 2017; Palencia, Rowcliffe, et al., 2021) or trends therein (Kéry & Andrew Royle, 2010). When individuals are unmarked (i.e., they cannot be uniquely identified), obtaining population size using CTs, has been achieved through different analytical frameworks, including time‐ or space‐to‐event models (Moeller et al., 2018), distance sampling (Howe et al., 2017), random encounter (and staying time) models (Nakashima et al., 2018; Rowcliffe et al., 2008), spatial capture–recapture (Chandler & Royle, 2013) and site‐structured abundance models (Kéry & Royle, 2016).

Broadly, these methods can be divided into two groups: (G1) those that estimate density from detection frequency and (G2) those that model animal counts as a function of an abundance and a detection parameter (i.e., detection probability or distance from an activity centre) that are jointly estimated (Loonam et al., 2021). Importantly, the state variables of interest (density D, and abundance N) are slightly different across methods. In G1, density D represents the expected number of individuals N at any instant in time and within the collective set of camera viewsheds (i.e. areas in front of the CT in which individuals can be detected) with a total area A. In G2, N refers to local or site‐abundance (henceforth “abundance”), the number of individuals available for detection during a specific survey duration and at a specific camera location. In this study, we will evaluate whether relative trends in abundance/density are consistent across these two paradigms of treating unmarked population counts. Specifically, we will focus on the random encounter model (REM) and N‐mixture model (NMM) (Royle, 2004) as representatives of G1 and G2, respectively. We choose these models, as they are the most widely adopted methods for estimating unmarked population size using CT data (Gilbert et al., 2020).

The REM estimates animal density from trapping rate (aggregated count across the survey period), the average size of the detection zone of the camera, and average movement speed of the population under study (Rowcliffe et al., 2008). Obtaining speeds of movement can be done by tagging individuals with GPS collars, but increases the cost of the study and usually leads to underestimation of movement (Rowcliffe et al., 2012; Sennhenn‐Reulen et al., 2017). However, a method to estimate the average speed of movement from CT pictures has recently been developed (Palencia, Fernández‐López, et al., 2021; Rowcliffe et al., 2016). REM makes the following assumptions: (i) detections are independent of each other, (ii) cameras are placed randomly relative to animal movement, (iii) individuals move independently of each other, and (iv) the populations under study are closed relative to the entire study area (i.e., no changes in overall population size within the survey period). The REM was found to be robust against violations in the independence of detections (Hayashi & Iijima, 2022), but not against non‐random placement of cameras relative to animal movement (Cusack et al., 2015). Moreover, REM is sensitive to biased movement speeds, as well as to the method used to estimate the range of the camera viewsheds (Santini et al., 2022). Nonetheless, Palencia, Rowcliffe, et al. (2021) obtained similar density estimates from REM compared to other methods representing G1. Finally, REM in its current form does not accommodate the modelling of spatial heterogeneity in density.

NMMs are hierarchical models that estimate the abundance at each camera (or site) based on counts from replicated surveys within the survey period rather than directly arriving at animal density for the collective set of camera viewsheds, as is done by REM. Consequently, the NMM requires that the study area is divided into discrete sites in order to infer abundance. The model assumes that (i) false‐positive detections do not occur, (ii) detections are independent of each other, (iii) each individual has the same probability of being detected, and (iv) the local population size does not change throughout the survey period. As abundances are typically biased when some or all of these model assumptions are violated (Barker et al., 2018; Fogarty & Fleishman, 2021; Kéry & Royle, 2016; Link et al., 2018), solutions have been proposed that involve elegant ways to relax these assumptions (Dail & Madsen, 2011; Martin et al., 2011). Here, we formulate an NMM for open populations (Dail & Madsen, 2011), with a beta‐Poisson detection process, building on ideas in Gomez et al. (2017) and Kéry and Royle (2016). Together, these adjustments accommodate changes in abundance between years and to some extent the occurrence of double counts (i.e., counting an individual twice during a survey). Furthermore, they allow the sharing of information on the detection process between commonly observed and rare species in a community (Gomez et al., 2017; Yamaura et al., 2011, 2012, 2016).

Since the state variables of interest (density D and abundance N) are different between REM and NMM, comparing these methods based on absolute estimates of their state variables would not yield valuable insights. Nevertheless, relative trends in their state variables should be largely consistent. However, rank‐order estimates between REM and NMM may diverge in some circumstances given their different treatments of animal counts. Therefore, the objective of this study is to evaluate if REM and NMM yield consistent (relative) trends: (i) between species, (ii) between years and (iii) across years obtained from annual density/abundance estimates. Specifically, we evaluate i–iii empirically by fitting REM and NMM to CT data of fox (*Vulpes vulpes*), red deer (*Cervus elaphus*), and wild boar (*Sus scrofa*) from a Mediterranean area in central Spain collected during five consecutive winter periods. We believe that this comparison is relevant given the importance of population trends in wildlife conservation and management (see Prowse et al., 2021 for a recent example), and because it compares methods representing two fundamentally different paradigms of unmarked abundance estimation.

## 2 MATERIALS AND METHODS

### 2.1 Study area

**FIGURE 1:**
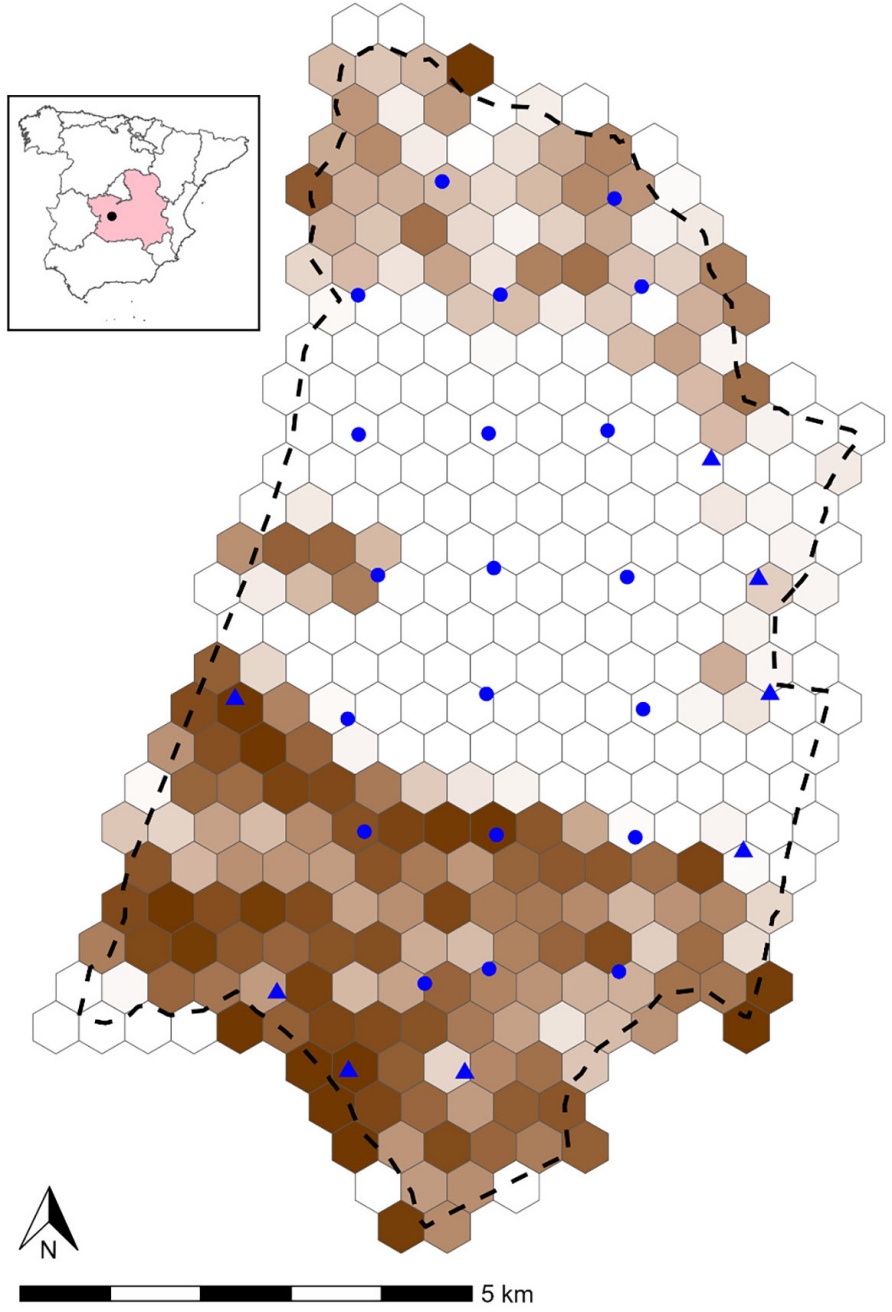
Map of the study area in Quintos de Mora with hexagonal grids. The colour scale represents the proportion of forest in each grid (white: low proportion; dark brown: high proportion). Camera locations that had a camera deployed every winter are indicated by dots, those with deployments only in the winter of 2019–20 by triangles. The inset map shows the study area within Spain and Castilla‐La Mancha (red).

The study area (longitudes: 4.148–4.048° W; latitudes: 39.342–39.460° N) is the Quintos de Mora National Reserve. It has a total surface area of 68.64 km^2^, and is located south of the Montes de Toledo. The centre of the area is characterised by an open savanna, while the mountain ranges in the north and south are dominated by Mediterranean shrubland and natural forests (Figure 1). In the savanna, the most predominant species is *Pinus pinea*, while forests and shrubland are mainly composed of a mixture of *Quercus coccifera*, *Quercus suber*, *Quercus ilex*, *Arbutus unedo*, *Erica* spp, and *Cistus* spp. Quintos de Mora has altitudes ranging from 720 to 1050 m above sea level. The climate is slightly continental, characterised by cold winters and hot summers. Quintos de Mora has an annual precipitation between 300 and 400 mm. The entire study area is fenced with fences impermeable to ungulates, such that the movement of wild boar and red deer in and out of the area should be limited. While these fences were not explicitly designed to be a movement barrier for fox, they may also hamper fox movement to some extent.

### 2.2 Camera trapping network and data

Within the study area, a CT network was deployed each winter from 2017–18 to 2021–22. Each of these winters, 20 cameras were installed at the intersections of a lattice grid (with a camera spacing of ~2 km), which were fixed across years (Figure 1; Table 1). During winter 2019–20, eight additional cameras were placed. This resulted in camera locations, which are, to the best of our knowledge, random relative to the movements of the three target species, i.e., fox, red deer and wild boar. These species were selected as they were the only ones that generated sufficient records for analysis by REM/NMM. As NMMs require data collected at discrete sites, we superimposed a hexagonal grid layer on the study area resulting in 336 grid cells of 0.2255 km^2^. This grid cell size trades off the possibility to capture fine‐scale spatial variation with the possibility of detecting individuals at multiple trapping locations. The number of trapping days varied between years. During the winters of 2017–18, 2018–19 and 2019–20, we used Bushnell Trophy Aggressor cameras, while Reconyx Hyperfire 2 and Browning Strike Force cameras were deployed during the winters of 2020–21 and 2021–22, respectively (Table 1). All cameras were mounted on trees ~50 cm above the ground, facing North and parallel to the ground. None of the cameras was baited to lure animals or was placed preferentially next to a trail. All cameras were set to be operative all day, to record a burst of consecutive photos (rapid fire) at each activation, and with the minimum triggering interval between activations. Timely check‐ups were performed to determine battery levels and to verify that the cameras were still operating. Groups of consecutive photos were aggregated into sequences, which were manually annotated and used for the analysis of density/abundance by both REM and NMM (Bollen et al., 2023).

**TABLE 1:** Description of the yearly camera trapping survey.

Year	Winter	Start date	End date	No. of days	No. of cams	Cam. Type
1	2017–18	28/09/2017	25/10/2017	28	19	Bushnell Trophy Aggressor
2	2018–19	29/11/2018	31/12/2018	33	19	
3	2019–20	18/09/2019	09/12/2019	83	28	
4	2020–21	01/10/2020	08/11/2020	39	19	Reconyx Hyperfire 2
5	2021–22	20/01/2022	16/02/2022	28	20	Browning Strike Force

### 2.3 Statistical models

We analysed CT data for three different species using the REM and an extension of the NMM. While the REM was applied to each of the species specific data independently for each year, a single spatially and temporally explicit beta‐Poisson NMM was fitted to the joint species data of all years in the study period.

#### 2.3.1 Random encounter model

We estimated animal density (individuals/km^2^) for each target species and year in our study period separately using REM. Fitting REM requires three sources of information: (i) encounter rate y/t, the rate at which individuals of a population come into contact with a CT, (ii) the radius r and angle θ of the camera viewsheds and (iii) animal movement speed v, which we obtained following Palencia, Rowcliffe, et al. (2021). First, we obtained the encounter rate of a target species by dividing the number of encounters y (i.e., total number of individuals from independent sequences of pictures of the species) by the total survey effort t (i.e., the sum of durations that each camera was active in the field). Note that we regarded pictures generated from each movement in and out of the camera viewshed as independent. Second, we estimated the effective radius $\hat{r}$ and angle $\hat{\theta }$ by applying distance sampling to recorded positions (radial distance and angle) of each individual entering a camera viewshed (Rowcliffe et al., 2011). Third, we approximated the movement speed of each individual by dividing its distance travelled through the camera viewsheds by the time it took (i.e. time between first and last photo). For each target species, we then identified its main behavioural modes and averaged across all speed measurements of the corresponding mode to obtain behaviour specific speeds for the population. Day range was obtained by summation of the products of behaviour specific speeds and the proportion of time spent on each behaviour (Palencia, Fernández‐López, et al., 2021). After obtaining encounter rate y/t, the radius r and angle θ of the camera viewsheds, and the day range (movement speed) v for each population, animal densities were estimated by:

$$D=\frac{y}{t}\cdot \frac{\pi }{v\cdot r\cdot \left(2+\theta \right)}$$

Finally, we calculated standard errors associated with density estimates for each population using the delta method (Seber, 1982).

#### 2.3.2 N‐mixture model

We modelled abundances for all target species and years in our study period using a single NMM. The NMM uses species counts, which are collected repeatedly in space and time, to model the expected number of individuals per site during a given survey period (abundance), knowing that some of the individuals that are present will not be detected (i.e., they do not show up in the species counts). However, the discrete sites (0.2255 km^2^) as we have defined them in section 2.2 are smaller than the typical home ranges of our target species. Hence, we cannot rule out that some individuals have been detected at multiple camera locations, violating the closure assumption. Thus, we interpreted abundances obtained from our NMM as relative abundances, i.e., the number of individuals that have used a site at least once during the survey period (henceforth “abundance” will refer to relative abundance).

We obtained species counts per survey day (24‐h) by summation across all the individuals of that species counted on sequences of pictures from that day. This yielded counts $y_{\text{sijt}}$ for species s=1,2,3 (fox, wild boar, red deer) at the subset of sites i=1,2,…,R that contained a CT during day j=1,2,…,J in year t=1,2,…,T. To correct for detection error NMM simultaneously estimates the detection probability (or rate) and the abundance of a species from $y_{\text{sijt}}$. We fitted our NMM to species counts $y_{\text{sijt}}$ within a Bayesian estimation framework using *Stan* (via the R package *cmdstanr*), a probabilistic programming language that enables Bayesian estimation through a dynamic Hamiltonian Monte Carlo (HMC) sampler (Carpenter et al., 2017). Specifically, we assumed $y_{\text{sijt}}$ to be *i.i.d*. Poisson random variables, such that the detection process is given by:

$$y_{\text{sijt}}|N_{\mathrm{sit}}\sim \text{Poisson}\left(N_{\mathrm{sit}}p_{\text{sijt}}\right),$$

where the mean is a product of the latent number of individuals of species s at site i during year *t* ($N_{\mathrm{sit}}$) and the species specific detection/trapping rate per camera per day ($p_{\text{sijt}}$). Note that by assuming a Poisson detection process, our NMM accommodates, to some extent, for double counts (i.e., it cannot account for individuals that are, on average, being detected >1 per survey day). We assumed that the trapping rate was constant across J days of year t and across R sites and that species specific detection rates $p_{\mathrm{st}}$ followed a beta distribution:

$$p_{\mathrm{st}}\sim \text{Beta}\left(p_{t}\tau ,\left[1-p_{t}\right]\tau \right),$$

where we parameterised $\text{Beta}\left(\alpha ,\beta \right)$, such that $\alpha =p_{t}\tau$ and $\beta =\left(1-p_{t}\right)\tau$. Under this parameterisation, $p_{t}$ and τ have a clear biological interpretation as the mean detection rate of all species in the community, and a measurement of similarity in species specific detection rates respectively (Dorazio et al., 2013). Furthermore, we modelled abundances $N_{\mathrm{sit}}$ as a Poisson process with mean $\lambda_{\mathrm{sit}}$:

$$N_{\mathrm{sit}}\sim \text{Poisson}\left(\lambda_{\mathrm{sit}}\right).$$

Without further restrictions, the likelihood of this model involves an infinite sum over $N_{\mathrm{sit}}$, which we needed to restrict in order to sample from it. Therefore, we set species specific finite upper bounds $K_{s}=\max\left(y_{\text{sijt}}\right)+100,\forall i=1,2,\ldots R;j=1,2,\ldots ,J\;\text{and}\;t=1,2,\ldots ,T$, which are much larger than the expected local population size ensuring that parameter estimates do not change appreciably beyond $K_{s}$. Moreover, we constructed the likelihood by marginalising over $N_{\mathrm{sit}}$'s with upper bound K given that *Stan* cannot sample discrete latent variables.

We defined two competing models, $M_{1}$ and $M_{2}$, for which the detection process is identical. Both models estimate the detection rate of the community as a smooth curve f across years:

$$\text{Logit}\left(p_{t}\right)=f\left(t\right).$$

However, the abundance process has an additional parameter in $M_{2}$ compared to $M_{1}$, capturing a linear trend in abundance across years for each species,

$$M_{1}:\mathrm{Log}\left(\lambda_{\mathrm{sit}}\right)=\beta_{s,0}+f_{s}^{\prime }\left(t\right)+f_{s}^{\text{HSGP}}\left({\mathrm{lon}}_{i},{\mathrm{lat}}_{i}\right)$$

$$M_{2}:\mathrm{Log}\left(\lambda_{\mathrm{sit}}\right)=\beta_{s,0}+\beta_{s,1}t+f_{s}^{\prime }\left(t\right)+f_{s}^{\text{HSGP}}\left({\mathrm{lon}}_{i},{\mathrm{lat}}_{i}\right)$$

where $\beta_{s,0}$ and $\beta_{s,1}$ represent species specific intercepts and slopes for the trends across years, $f_{s}^{\prime }$ models species specific smooth curves (trend noise), and $f_{s}^{\text{HSGP}}$ is a spatial random effect. Both f and $f_{s}^{\prime }$ use an exact Gaussian process (GP) (Golding & Purse, 2016; Williams & Rasmussen, 2006). For computational efficiency, we used the Hilbert‐space reduced rank Gaussian process (HSGP) approach to model $f_{s}^{\text{HSGP}}$ (Riutort‐Mayol et al., 2020; Solin & Särkkä, 2020). As the inclusion of species specific random effects markedly increases the number of parameters, possibly resulting in models that are too difficult to fit, we also tested non‐spatial versions of $M_{1}$ and $M_{2}$ omitting $f_{s}^{\text{HSGP}}$.

Prior specifications and goodness‐of‐fit diagnostics are detailed in Appendix A. We fitted all models using two parallel MCMC chains with 10,000 iterations, which included 5000 iterations that were discarded as burn‐in iterations; this always resulted in satisfactory convergence (Table A1), following the guidelines by Vehtari et al. (2021). After fitting $M_{1}$ and $M_{2}$ (and their non‐spatial versions), we performed a model selection by comparing their approximate leave‐one‐out expected log predictive densities (LOO‐ELPD) (Vehtari et al., 2017). For a comparison of the results from a beta‐binomial NMM and beta‐Poisson NMM, we refer to Appendix B.

### 2.4 Population trends

After model selection, we tested whether relative trends between species were consistent across the models by fitting a linear regression for yearly density (REM) versus abundance (NMM) estimates. We then compared temporal trends in density/abundance, obtained by REM and NMM in three ways. First, we computed the correlation between the ranks of relative trends between years in both methods using Spearman's rank correlation test. Next, we assessed the similarity of the trajectories of yearly growth rates, i.e., $x_{\mathrm{st}}/x_{s\left(t-1\right)}$ with $x=\left\{\begin{array}{c} \lambda \;\text{if}\;\mathrm{NMM}\\ D\;\text{if}\;\mathrm{REM} \end{array}\right.$ and also computed their Pearson correlations. Lastly, we compared slopes in linear trends across yearly densities/abundances. This is simply the estimated parameter $\hat{\beta_{s,1}}$ of $M_{2}$ for the NMM. However, to obtain this slope for REM, we needed to fit a linear regression line through estimates of yearly density post‐hoc (for reference, we also did this for the NMM). Finally, to assess the precision of parameter estimates, we compared the coefficient of variation (CV) between yearly abundance and density.

## 3 RESULTS

### 3.1 Trapping effort

Throughout the study period, we retain data from 4296 24‐h periods (fox and wild boar) and 2189 24‐h periods (red deer). This results in 2721, 520 and 226 observations of red deer, wild boar, and fox, respectively. The sampling period for fox and wild boar is extended relative to that of red deer, due to lower sample sizes in those species. Due to a defective camera, we retain data from only 19 CTs during the winters of 2017–18, 2018–19, and 2020–21.

### 3.2 Random encounter model

Mean annual densities estimated through REM lie between 0.41–0.73 individuals/km^2^ for fox, 5.34–7.14 for wild boar, and 25.06–46.63 for red deer (Table 2). We do not observe a consistent increase or decrease in yearly densities for any of the target species. Relevant interannual variation is observed in the encounter rate and day range in all of the species (Table 2). Only seven fox encounters are recorded during 2020, hence we could not estimate the fox density for that year.

**TABLE 2:** Values of the parameters of the estimated random encounter model (REM) for each population, where *y*/*t* is the encounter rate; *v*, the average distance travelled by an individual during a day (day range); *r*, the radius of detection; and *Ɵ*, the angle of detection.

Populations		Parameters				
Sp.	Year	*y*/*t* (ind/(cam·day))	*v* (km/day)	*r* (km)	*Ɵ* (rad)	Density (ind/km^2^)
Fox	1	0.042 (0.022)	13.920 (5.342)	0.0068 (0.0007)	0.733 (0.037)	0.56 (0.31)
	2	0.052 (0.021)	16.713 (5.212)	0.0064 (0.0006)	0.733 (0)	0.57 (0.3)
	3	0.075 (0.018)	20.532 (4.961)	0.0057 (0.0004)	0.733 (0)	0.73 (0.25)
	4	NA^a^	NA^a^	NA^a^	NA^a^	NA^a^
	5	0.069 (0.010)	21.225 (8.111)	0.0084 (0.0003)	0.960 (0.083)	0.41 (0.23)
Wild boar	1	0.191 (0.049)	6.840 (1.773)	0.0044 (0.0005)	0.733 (0.075)	7.14 (2.8)
	2	0.152 (0.053)	5.033 (1.634)	0.0057 (0.0005)	0.733 (0)	6.19 (1.92)
	3	0.177 (0.072)	8.823 (1.334)	0.0043 (0.0004)	0.733 (0.111)	5.34 (1.82)
	4	0.205 (0.029)	7.751 (1.483)	0.0048 (0.0003)	0.941 (0.126)	5.84 (3.32)
	5	0.158 (0.049)	5.638 (1.614)	0.0049 (0.0004)	0.733 (0)	6.55 (2.83)
Red deer	1	2.026 (1.061)	7.879 (1.279)	0.0059 (0.0002)	0.733 (0)	46. 63 (16.3)
	2	0.704 (0.120)	3.834 (0.827)	0.006 (0.0002)	0.733 (0)	34.87 (7.32)
	3	1.382 (0.182)	6.462 (0.511)	0.0053 (0.0001)	0.960 (0)	42.99 (9.21)
	4	0.670 (0.254)	4.020 (0.420)	0.0046 (0.0001)	0.960 (0)	44.92 (17.82)
	5	0.784 (0.117)	6.616 (0.984)	0.0050 (0.0001)	0.960 (0.174)	25.06 (8.48)

*Note*: Data represent means (± standard error).

^a^ Fox density in year 4 (2020–21) was not estimated due to the low sample size.

### 3.3 N‐mixture model

**FIGURE C1:**
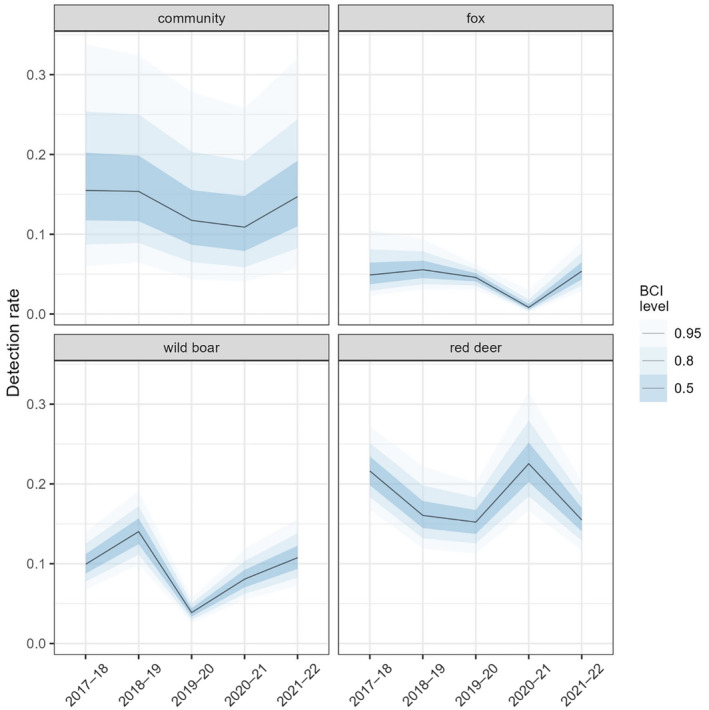
Posterior mean (and 50, 80 and 95% Bayesian credible intervals) of yearly detection rates for each species, obtained from $M_{1}$.

Model $M_{1}$ has the best predictive performance according to LOO‐ELPD, closely followed by $M_{2}$ (Table 3). However, the standard error on the ΔLOO‐ELPD between these models is substantial. Furthermore, LOO‐ELPD suggests that the spatial models are more consistent with the data than their non‐spatial counterparts. Both $M_{1}$ and $M_{2}$ fit the best to the fox data, followed by those of the red deer and finally the wild boar specific data (Table B2). For the remainder of the paper, all results from the NMM are generated based on the top‐ranking model ($M_{1}$). The posterior mean detection rate of the community decreases until 2020–21 and shows a slight increase from 2020–21 to 2021–22 (Figure C1). For all the years in our analysis, there is a fair amount of posterior uncertainty, judging from the 50%, 80%, and 95% Bayesian credible intervals (BCI) for the community detection rate. The fox specific detection rate mostly resembles the community detection rate in terms of its mean trend and posterior uncertainty (Figure C1). The mean detection rates for both wild boar and red deer are distinct from the trend of the community. BCIs are narrow for all years and species.

**TABLE 3:** Model comparison according to the leave‐one‐out expected log‐predictive densities (higher is better). Expected log‐predictive density, based on Leave‐one‐out (ELPD LOO).

Model	Eff. No. par.	ELPD LOO	SE (ELPD LOO)	ΔELPD LOO	SE (ΔELPD LOO)
$M_{1}$ (non‐spatial)	36	−5934.84	455.50	−40.04	12.99
$M_{2}$ (non‐spatial)	40	−5937.57	455.43	−42.77	12.91
$M_{1}$	77	−5894.79	453.97	0.00	0.00
$M_{2}$	81	−5896.64	453.91	−1.85	1.65

Abbreviation: Eff. No. par., effective number of parameters.

**FIGURE C2:**
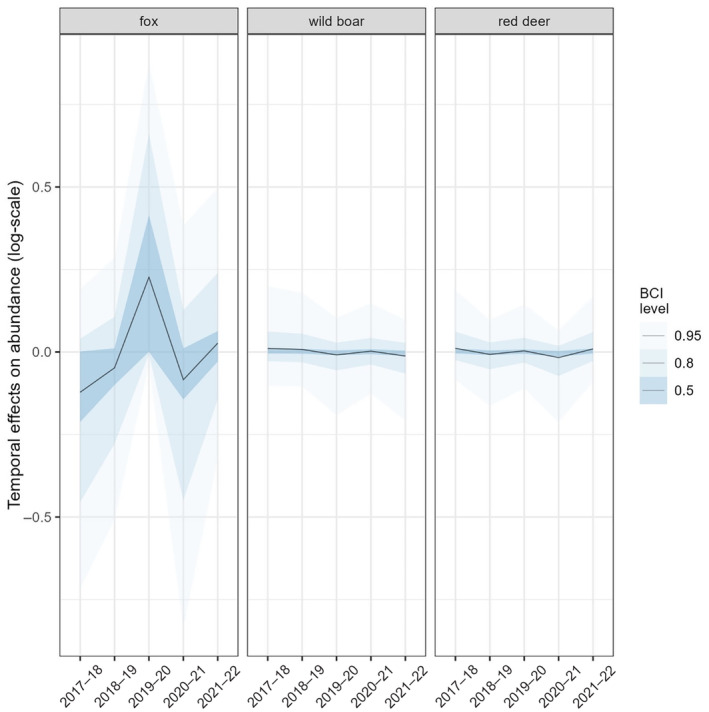
Posterior mean (and 50, 80 and 95% Bayesian credible intervals) of the smooth temporal variation (log‐scale) in abundance of each species, obtained from $M_{1}$.

**FIGURE C3:**
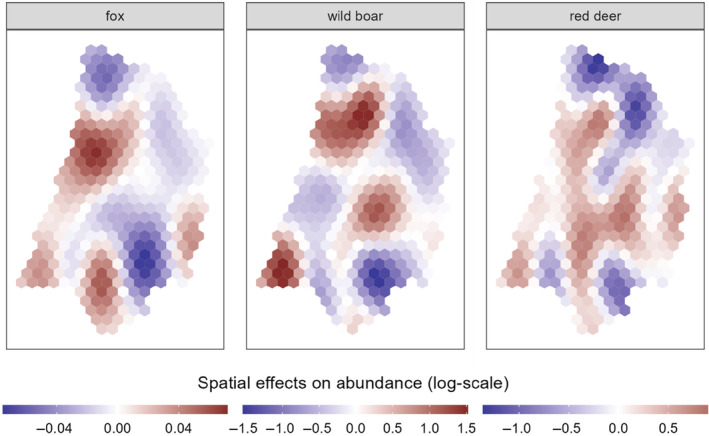
Posterior mean of the smooth spatial variation (log‐scale) in abundance of each species, obtained from $M_{1}$. For visual clarity, different scales are applied to each of the panels.

**FIGURE C4:**
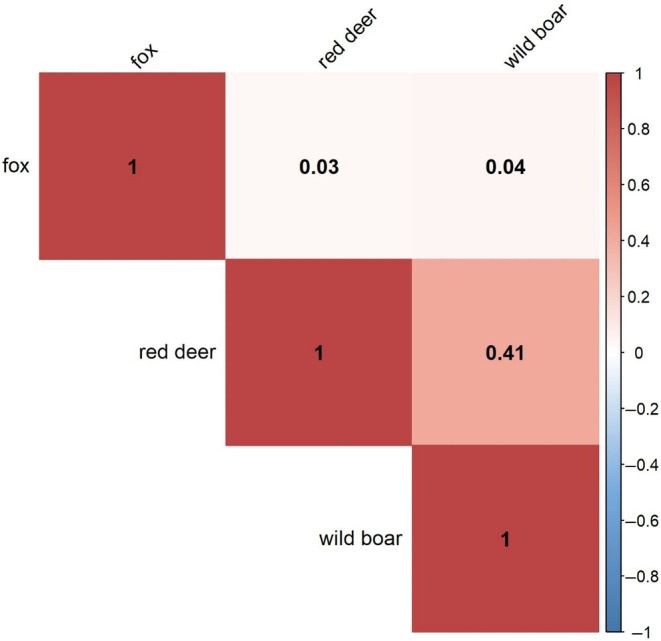
Correlations between the species specific HSGP coefficients $\left(\eta_{\mathrm{sm}}\right)$, obtained from $M_{1}$.

**FIGURE C5:**
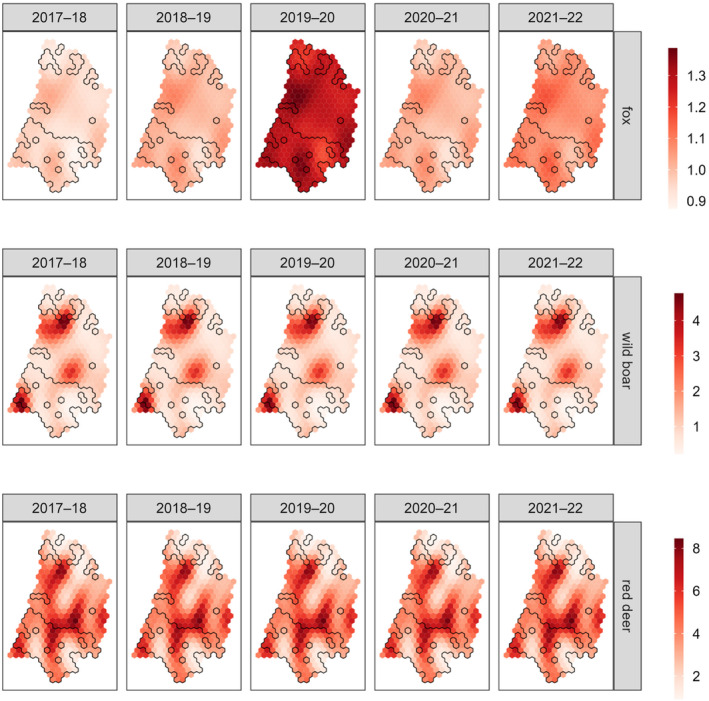
Posterior mean of yearly abundance for each species and each grid cell (0.2255 km^2^), obtained from $M_{1}$. Regions with more than 35% forest cover are enclosed by black lines. Heat scale: abundance – number of individuals that have used a grid cell at least once during the survey period.

**FIGURE C6:**
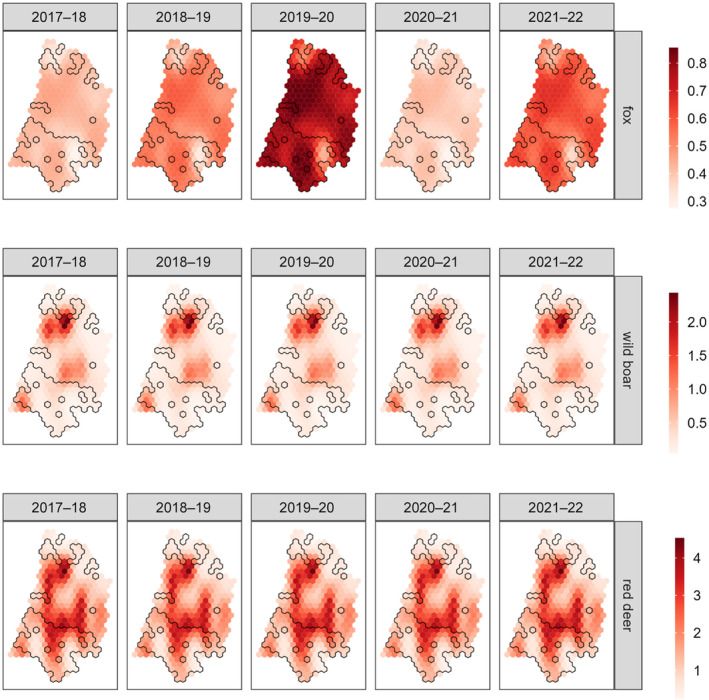
2.5th Percentile of posterior yearly abundance for each species, obtained from $M_{1}$. Regions with more than 35% of forest cover are enclosed by the black lines. Heat scale: abundance – number of individuals that have used a grid cell at least once during the survey period.

**FIGURE C7:**
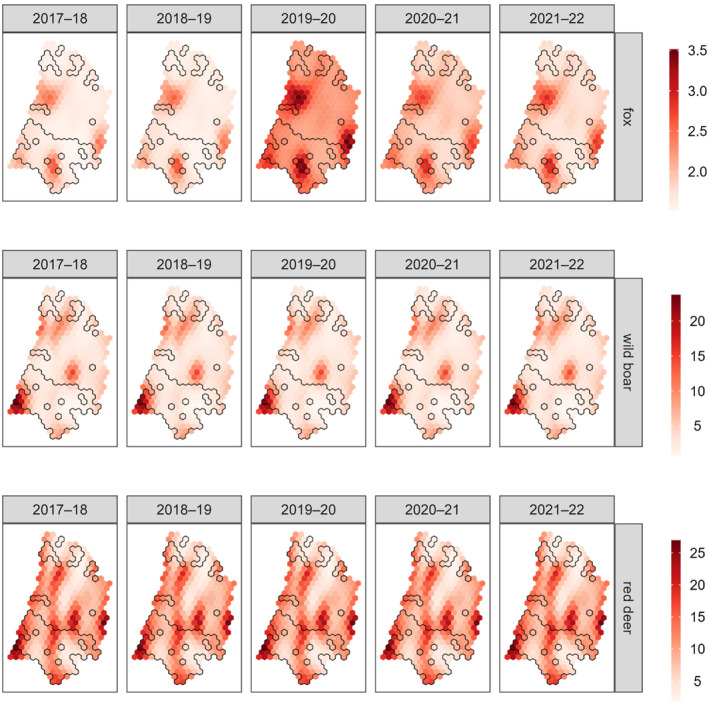
97.5th Percentile of posterior yearly abundance for each species, obtained from $M_{1}$. Regions with more than 35% of forest cover are enclosed by the black lines. Heat scale: abundance – number of individuals that have used a grid cell at least once during the annual survey period.

Smooth temporal effects reveal year‐to‐year fluctuations in abundance of similar magnitude, but different trends between red deer and wild boar (Figure C2). The yearly variation in fox is larger than both of these species and also has a different trend. Smooth spatial effects display different magnitudes for all species (Figure C3). The trend of spatial effects for foxes is not correlated with any of the spatial trends of other species (Figure C4). However, the spatial trends for red deer and wild boar are positively correlated. Together, relevant spatiotemporal variations in abundances are observed (Figures C5, C6, C7).

### 3.4 Population trends

**FIGURE 2:**
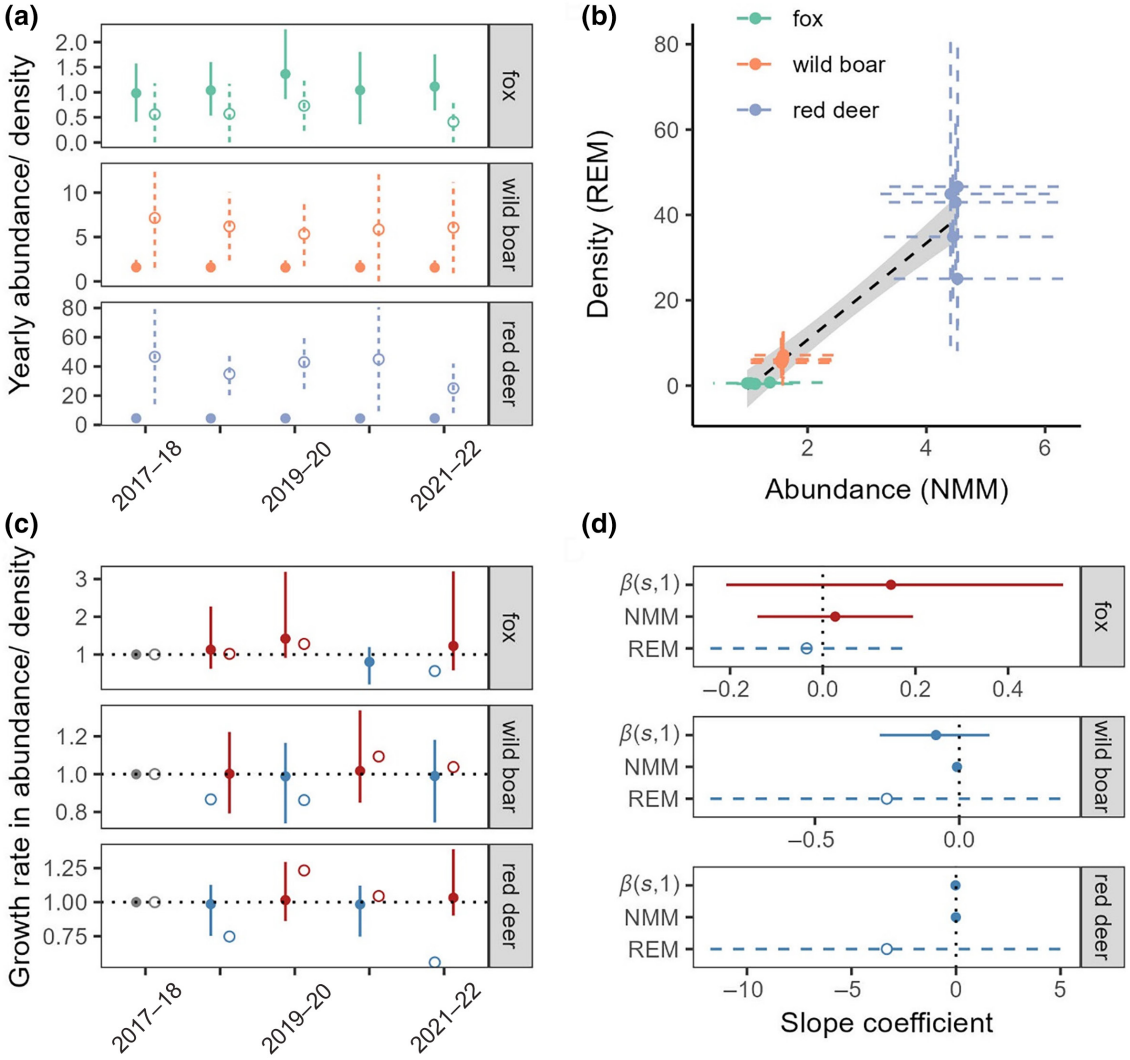
Consistency between population trends. (a) Mean ± 95% (B)CI abundances (NMM: closed circles ± full lines) and densities (REM: open circles ± dashed lines). (b) Linear trend ±95% CI bands for density (REM) versus abundance (NMM) estimates. 95% (B)CI are displayed for each pair of estimates. (c) Mean ± 95% (B)CI growth rates in abundance (NMM: closed circles ± full lines) and in density (REM: open circles. 95% CIs were not obtained due to a high error propagation using the delta method). (d) Mean ± 95% (B)CI slope coefficients for linear trends in yearly abundance (NMM) and density (REM) obtained by least squares regression. Linear trend in abundance captured by $\beta_{s,1}$ ($M_{2}$). No trend was visualised for $M_{1}$, as this model assumed that linear trends in abundances were absent. Colour scale – C: growth rate > 1 (red) or < 1 (blue), D: slope > 0 (red) or < 0 (blue). Fox density, and hence growth rate, in year 4 (2020–21) was not estimated by REM due to the low sample size.

Yearly densities (REM) and abundances (NMM) cannot be directly compared on their absolute scales (Figure 2c), yet they still contain important information on the consistency of species and/or year rankings across REM/NMM. The relationship between density (REM) and abundance (NMM) for the three species is captured well by a linear model (*R*‐squared: 0.9141; Figure 2b). Only the ranks of yearly densities/abundances for the entire community are significantly correlated between $M_{1}$ and REM (Table 4). However, both models produce similar trajectories in growth rates for fox, but not for wild boar and red deer (Figure 2c; Table 4). Interestingly, $M_{1}$ and REM are in agreement about the direction of linear trends in density/abundance estimates obtained post‐hoc, except for fox (Figure 2d). The 95% BCI of trend slopes from $M_{2}$ ($\beta_{s,1}$) overlap zero in all species. We did not attempt to compare the precisions of linear trends as they were obtained from values that are on substantially different scales.

**TABLE 4:** Correlation coefficients *ρ* and their significance for (i) a Spearman rank correlation test between yearly abundances (NMM) and yearly densities (REM) and (ii) a Pearson correlation test for growth rates in density of each species.

Species	Type	Statistic	*ρ*	*p*‐Value
Community	Spearman	30	.93	<.001
	Pearson	2.43	.63	.038
Fox	Spearman	6	.40	.750
	Pearson	5.38	.98	.120
Wild boar	Spearman	6	.70	.233
	Pearson	0.84	.51	.490
Red deer	Spearman	18	.10	.950
	Pearson	−0.34	−.23	.770

## 4 DISCUSSION

In this study, we compared trends (i) between species, (ii) between years and (iii) across years obtained from empirical populations of three target species based on two models: REM and NMM. We have focussed on trends rather than absolute state variables (density/abundance) for two main reasons. First, state variables are slightly different across REM (density) and NMM (abundance), and also rely on different characterisations of space and time (Gilbert et al., 2020; Loonam et al., 2021). Second, the use of absolute population size in conservation and management has been challenged (Morellet et al., 2007), particularly when these are obtained through NMMs (Dennis et al., 2015; Gilbert et al., 2020). Since all of our results are based on empirical data, i.e., the truth is not known, we will focus our discussion on the consistency and precision of estimated trends rather than discussing their accuracy. Moreover, we note that in some cases our study may be underpowered to detect (small) differences in abundance given the modest number of cameras that we deploy, i.e., 20 (+8 in 2019–20). Simulations may help to determine the number of cameras required to characterise trends in abundance (Ficetola et al., 2018).

As the NMM is very sensitive to model assumptions, we tried to control for two common sources of bias in abundance: accidental double counting of unmarked species by a Poisson detection process (Kéry & Royle, 2016; Link et al., 2018), and unmodelled heterogeneity (Duarte et al., 2018; Link et al., 2018; Veech et al., 2016) by the inclusion of several fixed (Figure 2d) and random effects (Figures C2 and C3). Our study used different CT types across years, which has likely induced variability in the probability of detecting individuals that cross a camera viewshed. To account for this source of variability and potentially other differences leading to interannual variation in detectability (Hofmeester et al., 2019), we have included temporal effects in NMM and estimated angles θ and radii r separately for each year when applying REM. Moreover, we attempted to control bias that may result from inaccurate estimates of movement speed in REM, by correcting for different movement speeds in the main behavioural modes of a species (Palencia, Fernández‐López, et al., 2021).

Although we warn users against using abundances from NMMs as absolute quantities (Barker et al., 2018), the yearly total abundances and densities retrieved from our NMM can be found in Table C1 for comparison with REM densities (Table 2). NMM and REM treat CT data differently (replicated counts vs. aggregated counts across the entire survey period). It is unclear how this impacts quantities derived from their state estimates, including relative trends, which is a study limitation. Regardless of the quantity of interest (either abundance or density), reaching a CV <0.25 is considered the minimum threshold for estimates to be useful for wildlife management (Skalski et al., 2005). The abundance estimates of our NMM meet this requirement across all species and years, while REM fails to deliver CV ≤ 0.25 for most species‐year combinations (Table C2). However, some caution is warranted as higher precisions could result from overconfidence, rather than from correctly characterised improvements (Goldstein & De Valpine, 2022). Possibly, the absence of movement parameters in the NMM, or the separation of model uncertainty over two subprocesses may lead to overconfidence in the precision of its abundances. On the contrary, underestimation of movement speed can lead to decreased precision of REM densities (Santini et al., 2022). Finally, weakly informative priors may have contributed to a lower CV (higher precision) in NMM abundances compared to densities from REM, which does not use priors.

Relative trends between species, based on species‐rankings of density/abundance, over a 5‐year period are consistent between NMM and REM (Figure 2b). A similar consistency in species‐rankings based on relative abundance indices from camera surveys and densities from faeces counts has been observed (Ferretti et al., 2023). Species specific spearman rank correlations between yearly NMM abundances and REM reveal that these models capture different relative trends between years. This is reinforced by the differential progressions of the yearly growth rates for wild boar and red deer (Figure 2c). The reduced consistency in yearly growth rates relative to species‐rankings between NMM and REM suggest that camera‐based estimators will converge on similar qualitative results when the range of variability is large (i.e. between‐species variability), but may not do so when the range of variability is smaller (i.e. between‐year variability). However, fox growth rates reveal a similar progression between the models and are highly correlated showing that camera‐based estimators may also yield consistent rank‐order patterns when variability is smaller (Figure 2c; Table 4). Simulation studies should be performed to identify the exact circumstances resulting in consistent rank‐order patterns between various camera‐based estimators, and those resulting in a lack of consistency. Linear trends in density/abundance across years, obtained post hoc, were in agreement only for two of the three species (Figure 2d). However, $M_{1}$ outperforming $M_{2}$ in terms of LOO‐ELPD suggests that these trends are stationary. This is supported by the BCIs of $\beta_{s,1}$ overlapping zero. The precision of trend estimates was higher in NMM compared to REM for all species. This could be a consequence of our NMM, but not our REM, explicitly modelling a temporal dependency between yearly abundances. Modelling temporal autocorrelation can indeed shrink outlying observations to the mean trend (Outhwaite et al., 2018; Williams & Rasmussen, 2006). The possibility to model (non)linear trends in abundance, rather than obtaining them post hoc, is a main advantage of the NMM as it allows for hypothesis‐testing through model selection (Table 3). This is also true for trends in space, which may help managers gain insight into the drivers of space use within a study area. In Quintos de Mora, for example, foxes seem to be more abundant in the west of the study area (Figure C5). Wild boar appear in three clusters, and red deer are clearly more abundant in the central savanna‐like landscape. All of the species seem to avoid the mountainous, forested areas in the north and south of the study area to some extent. Regardless of the exact spatial ecology of the target species in Quintos de Mora, being able to capture meaningful information on animal space use patterns in general is of high ecological importance and should be considered when choosing a modelling framework. Although REM for camera trapping data currently lacks the possibility to model spatial and/or temporal trends, it holds the potential to be extended to a fully spatiotemporal analysis tool following ideas in Jousimo and Ovaskainen (2016). Another possible avenue to capture spatial variation in density with REM, is to perform the analysis over a number of ecologically relevant strata. However, this may rapidly result in sample sizes that are too low to make meaningful inference when the number of strata increases or when the number of cameras available is limited.

In summary, we would advise practitioners against the use of NMMs when absolute densities/abundances are desired (although they may produce sensible estimates in some settings) and resort to REM or other methods instead (Efford & Fewster, 2013; Howe et al., 2017; Moeller et al., 2018; Nakashima et al., 2018). However, practitioners should be aware that all of these methods require auxiliary data and/or field procedures, some of which can be time‐consuming (Palencia, Rowcliffe, et al., 2021). Thus, when all that is needed are relative population trends, applying NMM may be faster, cheaper and can provide insight in spatiotemporal dynamics of abundance. To bridge this gap we encourage the further development and adoption of spatiotemporally explicit versions of REM for camera trapping studies. Finally, it appears that NMM and REM converge on similar relative trends when the range of variability is large, but may not do so when the range of variability is smaller. We advise that researches take this into account, when they want to directly compare the qualitative results from different camera‐based estimators or perform meta‐analyses thereof. Since the truth in our study is unknown, we cannot draw conclusions about the accuracy of these trends. Hence, we encourage future research that evaluates the accuracy of relative trends in a simulation setting.

## AUTHOR CONTRIBUTIONS

**Martijn Bollen:** Conceptualization (lead); formal analysis (equal); funding acquisition (equal); methodology (equal); visualization (lead); writing – original draft (lead); writing – review and editing (equal). **Pablo Palencia:** Conceptualization (supporting); formal analysis (equal); methodology (equal); validation (equal). **Joaquín Vicente:** Conceptualization (supporting); project administration (lead); validation (equal); writing – review and editing (equal). **Pelayo Acevedo:** Conceptualization (supporting); project administration (lead); validation (equal); writing – review and editing (equal). **Lucía Del Río:** Data curation (lead). **Thomas Neyens:** Conceptualization (supporting); methodology (supporting); supervision (equal); writing – review and editing (equal). **Natalie Beenaerts:** Conceptualization (supporting); supervision (lead); writing – review and editing (equal). **Jim Casaer:** Conceptualization (lead); methodology (supporting); supervision (equal); writing – review and editing (equal).

## CONFLICT OF INTEREST STATEMENT

The authors declare no conflicts of interest.

## Figures and Tables

**FIGURE B1:**
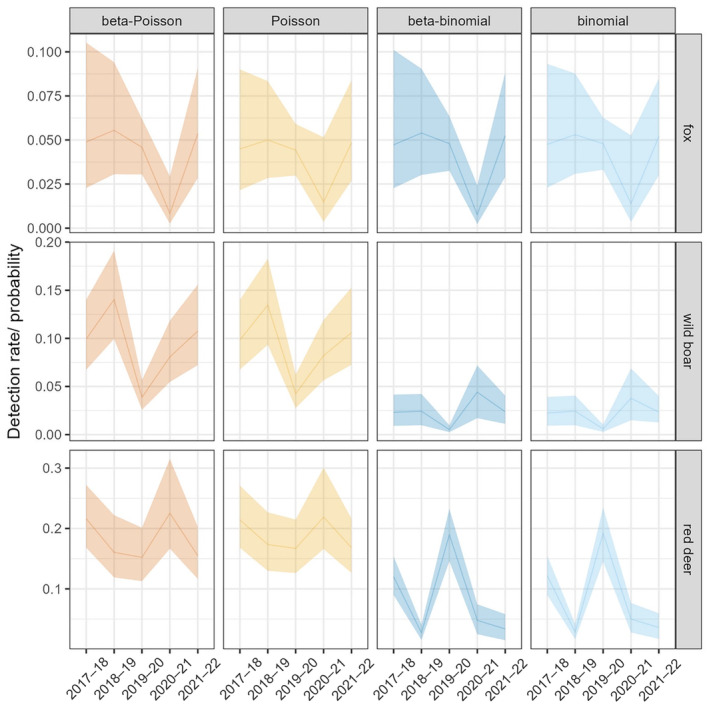
Posterior mean (and 95% Bayesian credible intervals) of yearly detection rate/probability for each species, obtained from $M_{1}$, a beta‐binomial version of $M_{1}$, and single‐species versions of $M_{1}$ with a Poisson or a binomial observation process.

**FIGURE B2:**
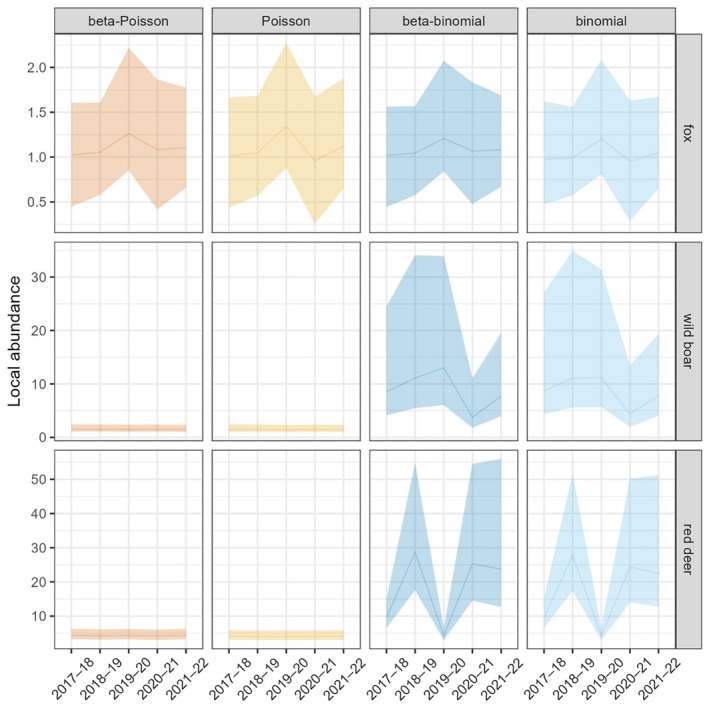
Posterior mean (95% Bayesian credible intervals) of yearly abundances for each species, obtained from $M_{1}$, a beta‐binomial version of $M_{1}$, and single‐species versions of $M_{1}$ with a Poisson or a binomial observation process.

## Data Availability

The data that support the findings of this study are openly available in the Dryad digital repository at https://doi.org/10.5061/dryad.g1jwstqwd.

## APPENDIX A PRIOR SPECIFICATION, MODEL CONVERGENCE AND GOODNESS‐OF‐FIT OF THE N‐MIXTURE MODELS

### A.1

For the beta‐Poisson N‐mixture models, which are described in the main paper, we used mostly vaguely informative priors of the normal, studentt or gamma distribution. Only for the length scale parameter of the GPs we used distributions that avoid values near zero (i.e. inverse gamma), or that avoid both values near zero and larger values (i.e. generalized inverse Gaussian). More specifically, we specified a $\text{gamma}\left(5,0.1\right)$ for the dispersion parameter τ. For regression parameters $\beta_{s,0},\beta_{s,1}$, we used $\text{student}\;t\left(3,0,5\right)$ and $\text{normal}\left(0,1\right)$ priors respectively. We used $\text{half normal}\left(0,1\right)$ priors, and an inverse gamma $\mathrm{IG}\left(11,\;4\right)$ prior for respectively the marginal standard deviations $\left\{\sigma_{f_{s}},\sigma_{f_{s}^{\prime }}\right\}$ and the length scales $\left\{\rho_{f},\rho_{f_{s}^{\prime }}\right\}$, the hyperparameters of the GPs. For the same hyperparameters of the HSGP, i.e., $\sigma_{f_{s}^{\text{HSGP}}}$ and $\rho_{f_{s}^{\text{HSGP}}}$, we used a non‐negative $\text{student}\;t^{+}\left(3,0,5\right)$ and a generalised inverse Gaussian $\mathrm{GIG}\left(3,11,0.01\right)$ prior. For the GPs coefficients $\left\{\eta_{f},\eta_{f_{s}^{\prime }}\right\}$, we used $\text{normal}\left(0,1\right)$ priors. We specified a multivariate normal distribution for the HSGP coefficients $\eta_{f_{s}^{\text{HSGP}}}$, with a zero‐mean and variance–covariance matrix Σ. For numerical efficiency, we decomposed Σ into its Cholesky factors (i.e., $\Sigma =L\cdot \sigma_{\eta }\cdot L^{\prime }$), with $L\sim \mathrm{LKJ}\left(4\right)$ and $\sigma_{\eta }\sim \text{half normal}\left(0,1\right)$. Here we also show diagnostics on the convergence of the MCMC and on the exploration of the posterior for the most important parameters of $M_{1}$ (Table A1).

Finally, to check the goodness‐of‐fit, we calculated $\chi^{2}$ – discrepancies, relative to the expected counts under $M_{1}$ and $M_{2}$, for the actual data $\left(T\right)$ and replicated data (i.e., simulated from the model; $T^{\left(s\right)}$). For a satisfactory model fit, the $\chi^{2}$ – discrepancies calculated from replicated data should align with those obtained from actual data, i.e., $\hat{C}=T/T^{\left(s\right)}\approx 1$ (Table A2).

**TABLE A1:** Diagnostics assessing the convergence of the MCMC sampler (Rhat), and the efficiency of the posterior exploration in the bulk and tail of the distribution (ESS bulk, ESS tail) for $M_{1}$.

Parameter	Rhat	Rhat (2.5th)	Rhat (97.5th)	ESS bulk	ESS bulk (2.5th)	ESS bulk (97.5th)	ESS bulk	ESS tail (2.5th)	ESS tail (97.5th)
τ	1.0002	1.0002	1.0002	3755	3755	3755	2467	2467	2467
$\beta_{s,0}$	1.0008	0.9998	1.0017	3091	2221	4279	3296	2827	3583
$\sigma_{f_{s}}$	1.0018	1.0018	1.0018	2307	2307	2307	2946	2946	2946
$\sigma_{f_{s}^{\prime }}$	1.0072	1.0003	1.0168	654	383	826	1846	1571	2230
$\sigma_{f_{s}^{\text{HSGP}}}$	1.0004	1.0000	1.0011	3253	1711	4158	3717	3300	4332
$\rho_{f}$	1.0000	1.0000	1.0000	4214	4214	4214	3199	3199	3199
$\rho_{f_{s}^{\prime }}$	1.0010	1.0010	1.0010	8007	8007	8007	3455	3455	3455
$\rho_{f_{s}^{\text{HSGP}}}$	1.0009	1.0005	1.0012	2628	2016	3263	3048	2653	3346
$\eta_{f}$	1.0014	1.0008	1.0022	3842	3324	4225	3541	3127	3901
$\eta_{f_{s}^{\prime }}$	1.0009	0.9999	1.0032	5440	1775	6794	3876	2689	4425
$\eta_{f_{s}^{\text{HSGP}}}$	1.0006	0.9998	1.0020	6888	4014	8698	3948	3197	4544
$\sigma_{\eta }$	1.0004	0.9999	1.0008	3284	1853	4241	3589	3287	4095

*Note*: For each diagnostic, the mean, 2.5th and 97.5th percentiles are displayed. Note that we only display parameters that have a prior distribution in $M_{1}$.

Abbreviations: ESS, effective sample size (in the tail or bulk of the distribution); MCMC, Markov chain Monte Carlo; Rhat, potential scale reduction factor.

**TABLE A2:** Posterior means and 95% credible intervals for species specific overdispersion diagnostic $\left(\hat{C}\right)$ obtained from $M_{1}$ and $M_{2}$.

Species	Model	Mean	2.50%	97.50%
Fox	$M_{1}$	0.8454	0.6933	1.0264
	$M_{2}$	0.8464	0.697	1.0259
Wild boar	$M_{1}$	2.3601	2.0432	2.7045
	$M_{2}$	2.3615	2.0479	2.7162
Red deer	$M_{1}$	1.5102	1.3825	1.6444
	$M_{2}$	1.5118	1.3872	1.6502

## APPENDIX B RESULTS FOR ALTERNATIVE OBSERVATION MODELS OF $M_{1}$

### B.1

In the main paper, we formulated $M_{1}$ as a beta‐Poisson NMM. Here we show the most important findings obtained by changing the observation process of $M_{1}$ to either a Poisson, beta‐binomial, or binomial distribution. Note that the beta‐binomial NMM models species specific data jointly, just like the beta‐Poisson NMM, while the Poisson NMM and binomial NMM need to be fitted to data of each species separately. In our study, a joint approach using a beta distribution did not qualitatively change the inference from NMM (Figures B1 and B2), but yielded speedups in computation time (Table B1). Specifying a (beta‐)Poisson instead of a (beta‐)binomial model was effective against inflation of abundance for wild boar and red deer (Figure B2), and has probably helped to control bias from accidental double counting.

**TABLE B1:** Time (in seconds) needed to fit $M_{1}$, a beta‐binomial version $M_{1}$, and single‐species versions of $M_{1}$ with a Poisson or a binomial observation process for all species in the community.

Model	Walltime in sec.	Fold change
Beta‐Poisson	10,472	1.00
Poisson	13,052	1.25
Beta‐binomial	19,464	1.86
Binomial	24,068	2.30

**TABLE B2:** Posterior means and 95% credible intervals for species‐specific overdispersion diagnostic $\left(\hat{C}\right)$ obtained from $M_{1}$, a beta‐binomial version $M_{1}$, and single‐species versions of $M_{1}$ with a Poisson or a binomial observation process.

Model	Species	Mean	2.50%	97.50%
Beta‐Poisson	Fox	0.8454	0.6933	1.0264
	Wild boar	2.3601	2.0432	2.7045
	Red deer	1.5102	1.3825	1.6444
Poisson	Fox	0.8554	0.7062	1.0433
	Wild boar	2.3591	2.0544	2.7075
	Red deer	1.5041	1.3772	1.6386
Beta‐binomial	Fox	0.9000	0.7559	1.0724
	Red deer	2.4171	2.1126	2.7658
	Red deer	1.6648	1.5302	1.8115
Binomial	Fox	0.8970	0.7537	1.0667
	Wild boar	2.4155	2.1182	2.7570
	Red deer	1.6604	1.5361	1.7965

## APPENDIX C ADDITIONAL FIGURES AND TABLES FOR $M_{1}$

### C.1

In the main paper, we do not include visualisations for the detection rates, temporal and spatial random effects for the beta‐Poisson NMM. Here we show these results based on $M_{1}$, and we also include maps for the 2.5th and 97.5th percentiles of abundances. Furthermore, we give an overview of the posterior total abundance and density per species per year derived from $M_{1}$ (Table C1). We computed the total abundance, and density based on $M_{1}$ by: (i) calculating the expected number of individuals $E\left(N_{\mathrm{sit}}\right)$ of species s at site i during year t, (ii) summing these expectations over all sites, such that $E\left(N_{\mathrm{st}}\right)=\sum_{i=1}^{R}E\left(N_{\mathrm{sit}}\right)$, and (iii) scaling them by the total surface area in the grid layer (i.e., the product of R, the number of sites, and $A_{i}$, the area (in km^2^) per site), hence $E\left(D_{\mathrm{st}}\right)=E\left(N_{\mathrm{st}}\right)/\left(R\cdot A_{i}\right)$. Finally, we show comparisons of the coefficient of variation (CV) between abundances from $M_{1}$ and densities from REM (Table C2).

**TABLE C1:** Estimated beta‐Poisson N‐mixture model parameter values for each population, where the abundance $\lambda_{\mathrm{st}}$ is estimated directly, and the total abundance $E\left(N_{\mathrm{st}}\right)$ and density $E\left(D_{\mathrm{st}}\right)$ are derived from the number of sites R, and their respective surface area $A_{i}$.

Populations		Parameters		
Sp.	Year	Abundance (mean ind/site)	Total abundance (ind)	Density (ind/km^2^)
Fox	1	1.02 (0.29)	16.4 (5.51)	3.83 (1.29)
	2	1.06 (0.26)	17.8 (4.25)	4.16 (0.99)
	3	1.34 (0.36)	44.86 (8.16)	6.86 (1.25)
	4	1.09 (0.38)	19.45 (8.21)	4.54 (1.92)
	5	1.13 (0.29)	22.12 (5.47)	4.91 (1.21)
Wild boar	1	1.58 (0.36)	31.39 (5.01)	7.33 (1.17)
	2	1.57 (0.36)	29.86 (4.57)	6.97 (1.07)
	3	1.55 (0.35)	40.39 (7.74)	6.18 (1.18)
	4	1.57 (0.41)	29.88 (5.34)	6.97 (1.25)
	5	1.55 (0.37)	27.46 (4.59)	6.09 (1.02)
Red deer	1	4.45 (0.77)	80.3 (9.55)	18.74 (2.23)
	2	4.36 (0.76)	73.81 (11.17)	17.23 (2.61)
	3	4.41 (0.77)	121.71 (18.03)	18.61 (2.76)
	4	4.32 (0.76)	73.13 (11.57)	17.07 (2.7)
	5	4.43 (0.77)	84.76 (11.48)	18.79 (2.54)

*Note*: Data represent posterior means (± posterior standard deviations).

**TABLE C2:** Estimated model parameter values for each population.

Populations			Parameters			
Sp.	Year	Model	Mean (SD)	CV	2.5%–97.5%	(B)CI width
Fox	1	NMM	1.02 (0.29)	0.29	0.45–1.61	1.16
		REM	0.56 (0.31)	0.55	0–1.18	1.18
	2	NMM	1.06 (0.26)	0.24	0.58–1.61	1.03
		REM	0.57 (0.3)	0.53	0–1.17	1.17
	3	NMM	1.34 (0.36)	0.27	0.85–2.22	1.37
		REM	0.73 (0.25)	0.34	0.23–1.23	1
	4	NMM	1.09 (0.38)	0.35	0.42–1.87	1.45
		REM	NA	NA	NA	NA
	5	NMM	1.13 (0.29)	0.26	0.66–1.78	1.12
		REM	0.41 (0.23)	0.56	0–0.87	0.87
Wild boar	1	NMM	1.58 (0.36)	0.23	1.07–2.42	1.35
		REM	7.14 (2.8)	0.39	1.54–12.74	11.2
	2	NMM	1.57 (0.36)	0.23	1.06–2.39	1.32
		REM	6.19 (1.92)	0.31	2.35–10.03	7.68
	3	NMM	1.55 (0.35)	0.23	1.04–2.35	1.31
		REM	5.34 (1.82)	0.34	1.7–8.98	7.28
	4	NMM	1.57 (0.41)	0.26	1.05–2.38	1.33
		REM	5.84 (3.32)	0.57	0–12.48	12.48
	5	NMM	1.55 (0.37)	0.24	1.02–2.33	1.31
		REM	6.06 (2.57)	0.42	0.92–11.2	10.28
Red deer	1	NMM	4.45 (0.77)	0.17	3.26–6.28	3.01
		REM	46.63 (16.3)	0.35	14.03–79.23	65.2
	2	NMM	4.36 (0.76)	0.17	3.14–6.12	2.98
		REM	34.87 (7.32)	0.21	20.23–49.51	29.28
	3	NMM	4.41 (0.77)	0.17	3.23–6.24	3
		REM	42.99 (9.21)	0.21	24.57–61.41	36.84
	4	NMM	4.32 (0.76)	0.18	3.06–6.05	2.99
		REM	44.92 (17.82)	0.4	9.28–80.56	71.28
	5	NMM	4.43 (0.77)	0.17	3.23–6.29	3.05
		REM	25.06 (8.48)	0.34	8.1–42.02	33.92

*Note*: Means ± standard deviations in abundance $\lambda_{\mathrm{st}}$ for NMM, and in density abundance $D_{\mathrm{st}}$ for REM.
